# Addendum: Li, Y.; Shi, X.; Wei, L.; Zou, J.; Chen, F. Assigning Main Orientation to an EOH Descriptor on Multispectral Images. Sensors 2015, 15, 15595–15610.

**DOI:** 10.3390/s151229776

**Published:** 2015-11-30

**Authors:** Yong Li, Xiang Shi, Lijun Wei, Junwei Zou, Fang Chen

**Affiliations:** 1 Beijing University of Posts and Telecommunications, School of Electronic Engineering, Rd. Xitucheng 10#, Beijing 100876, China; 2013140640@bupt.edu.cn (X.S.); weilijun@bupt.edu.cn (L.W.); buptzjw@bupt.edu.cn (J.Z.); 2 Beijing Anzhen Hospital, Capital Medical University and Beijing Institute of Heart Lung and Blood Vessel Disease, Beijing 100029, China; azchenfang@163.com

The authors wish to update the Acknowledgments section in their paper published in Sensors [1], doi:10.3390/s150715595, http://www.mdpi.com/1424-8220/15/7/15595.
